# Diabetes increases risk of lumbar spinal fusion complications: association with altered structure of newly formed bone at the fusion site

**DOI:** 10.1093/jbmrpl/ziae053

**Published:** 2024-04-15

**Authors:** Claire Wilson, Piotr J Czernik, Hossein Elgafy, Sadik Khuder, Kevin Serdahely, Andrea Rowland, Beata Lecka-Czernik

**Affiliations:** Department of Orthopedic Surgery, University of Toledo, Toledo, OH 43614, United States; Department of Orthopedic Surgery, University of Toledo, Toledo, OH 43614, United States; Department of Orthopedic Surgery, University of Toledo, Toledo, OH 43614, United States; Department of Medicine, University of Toledo, Toledo, OH 43614, United States; Department of Orthopedic Surgery, University of Toledo, Toledo, OH 43614, United States; Department of Medicine, University of Toledo, Toledo, OH 43614, United States; Department of Orthopedic Surgery, University of Toledo, Toledo, OH 43614, United States; Center for Diabetes and Endocrine Research, College of Medicine & Life Sciences, University of Toledo, Toledo, OH 43614, United States

**Keywords:** diabetes, lumbar spine fusion, spine degenerative disease, adjacent segment disease, non-union revision

## Abstract

Diabetes predisposes to spine degenerative diseases often requiring surgical intervention. However, the statistics on the prevalence of spinal fusion success and clinical indications leading to the revision surgery in diabetes are conflicting. The purpose of the presented retrospective observational study was to determine the link between diabetes and lumbar spinal fusion complications using a database of patients (*n* = 552, 45% male, age 54 ± 13.7 years) residing in the same community and receiving care at the same health care facility. Outcome measures included clinical indications and calculated risk ratio (RR) for revision surgery in diabetes. Paravertebral tissue recovered from a non-union site of diabetic and nondiabetic patients was analyzed for microstructure of newly formed bone. Diabetes increased the RR for revision surgery due to non-union complications (2.80; 95% CI, 1.12–7.02) and degenerative processes in adjacent spine segments (2.26; 95% CI, 1.45–3.53). In diabetes, a risk of revision surgery exceeded the RR for primary spinal fusion surgery by 44% (2.36 [95% CI, 1.58–3.52] vs 1.64 [95% CI, 1.16–2.31]), which was already 2-fold higher than diabetes prevalence in the studied community. Micro-CT of bony fragments found in the paravertebral tissue harvested during revision surgery revealed structural differences suggesting that newly formed bone in diabetic patients may be of compromised quality, as compared with that in nondiabetic patients. In conclusion, diabetes significantly increases the risk of unsuccessful lumbar spine fusion outcome requiring revision surgery. Diabetes predisposes to the degeneration of adjacent spine segments and pseudoarthrosis at the fusion sites, and affects the structure of newly formed bone needed to stabilize fusion.

## Graphical Abstract

**Graphical Abstract:**
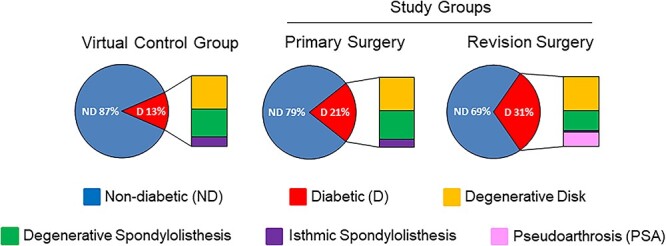

## Introduction

Lower back degenerative spinal diseases and spinal fusion surgical interventions are on the rise and coincide with an increased prevalence of diabetes, which affects over 37 million Americans, approximately 11.3% of the population.^1^^,^^2^ Diabetes is known to have a widespread effects on an individual’s musculoskeletal health, including increased bone fragility, osteoarthritis, and spine diseases.^3-7^ There is a strong association between diabetes and intervertebral disc degeneration.^8-11^ Both insulin-dependent (type 1) and insulin-independent (type 2) diabetes are risk factors for developing lumbar degenerative disease, with severity correlated with duration of the diabetic disease.^6^

Spinal fusion surgery using pedicle screws is a well-accepted surgical procedure for the treatment of degenerative lumbar conditions. Revision surgery is performed in 9%–45% of patients and the risk increases with increasing time following initial surgery.^12^^,^^13^ While technical error, infection, or postoperative complications indicate early revision, later revision surgery is often indicated for pseudarthrosis (non-union) or adjacent segment disease (ASD).^14-16^ Diabetes has been shown to be associated with worse spinal fusion surgery outcomes and higher complication rates, including higher rate of postsurgical infections and deep venous thrombosis (DVT)^17^; however, this analysis did not specify whether the surgery was causative for the increase in DVT development or whether it was unrelated to surgery and reflected predisposition to develop DVT in diabetes. Nevertheless, other studies showed that diabetic patients have significantly worse improvement in preoperative symptoms including leg pain and numbness, often associated with DVT, as compared with nondiabetic patients.^18-20^ Furthermore, poor glycemic control correlates with higher inpatient mortality and higher rate of perioperative complications.^21^ However, available retrospective analyses provide conflicting results on the success of lumbar spinal fusion surgery in diabetes with respect to postoperative complications associated with progressing ASD and affected new bone formation stabilizing fusion of vertebrae. A few studies performed in Chinese, Japanese, and Iranian populations showed no association between diabetes and the development of ASD following posterior lumbar fusion,^22-25^ whereas other Chinese studies reported significantly increased risk for ASD and reoperation rate in diabetic patients.^15^^,^^26^ In addition, diabetes was associated with a higher incidence of screw loosening, indicating pseudoarthrosis,^13^^,^^27^ which would be consistent with affected bone formation and compromised bone quality characteristic of diabetic bone.^3^^,^^5^^,^^28^ Thus, geographic, demographic and ethnic differences between studied cohorts may be significant confounding factors responsible for these conflicting results.

The current study was conducted to determine the association between diabetes and lumbar spinal fusion complications requiring surgical intervention. A retrospective analysis was conducted using a database of patients residing in the same community and receiving care at the same medical facility and was accompanied with a study of microarchitecture of newly formed bone residing in paravertebral tissue isolated from the spinal fusion bed during revision surgery. The analysis shows that diabetes increases the risk of development of ASD and pseudoarthrosis associated with structural defects of newly formed bone.

## Materials and methods

### Cohort characterization

The study included patients with lumbar spinal fusion (International Classification of Diseases, Tenth Revision, Clinical Modification [ICD-10-CM] classification M43.26), either as a primary procedure or as a revision of previous spinal fusion surgery including patients with multiple revisions. The analyzed patient population had been under the care of a single spine surgeon at an academic medical center located in Lucas County, northwest Ohio. The surgeries were performed between June 2009 and December 2017. The medical records were obtained under the approved protocol (IRB #202392) by the Institutional Human Research Protection Review Board. Information was collected through the electronic medical records and included patient age, sex, BMI, diabetic status indicated by blood levels of glycated hemoglobin (HbA1c) higher than 6.5% and/or use of antidiabetic medications, smoking history, alcohol use, and the date of a patient’s surgery.

### Inclusion criteria

Inclusion criteria were a minimum of 18 years of age, being a resident of Lucas County, and having undergone primary or revision spinal surgery. Procedures included fusion, decompression, laminectomy, and posterolateral and transforaminal lumbar interbody fusion involving a range of 1 to 8 levels of the lumbar spine. Patients who underwent spinal surgery due to trauma or infection were excluded from the analysis.

### Indications for the initial spinal fusion surgery and for revision surgery

The spinal fusion patient cohort had been stratified based on indications for surgery (ICD-10 classification: M43.26) and diabetes status. These indications included degenerative conditions (with the following terms: degenerative disc, degenerative prolapsed disc, degenerative spondylolisthesis, spondylolysis, degenerative scoliosis, isthmic spondylolisthesis, and pars defect) and pseudoarthrosis.

Successful fusion was assessed radiographically and included standing anteroposterior and lateral radiographs and CT scans. Radiographic fusion within the intervertebral disc space was identified by the formation of trabecular bony bridging, incorporation of the interbody graft into the adjacent endplate, and lack of segmental motion on flexion–extension lateral radiographs. Posterolateral fusions were evaluated according to the method popularized by Lenke et al,^29^ as follows: Grade A, definitely solid with bilateral stout fusion masses present; Grade B, probably solid with a unilateral stout fusion mass and a contralateral thin fusion mass; Grade C, probably not solid with a thin unilateral fusion mass and a probable pseudarthrosis on the contralateral side; and Grade D, definitely not solid with thin fusion masses bilaterally with obvious pseudarthrosis or bone graft dissolution bilaterally. Grades A and B were considered fused and Grades C and D were considered not fused. Overall fusion ratings were based on assessment of all levels treated, where the lowest rating at any individual level was considered the overall rating. There was a tendency for pseudoarthrosis to occur in long-segment, especially at L5–S1, versus short-segment fusion. Also, the risk of pseudoarthrosis was lower when interbody fusion was done in association with posterolateral fusion versus posterolateral fusion alone.

### Sources of national and Ohio state information on spinal fusion and diabetes prevalence

Data for the national prevalence of diabetes were compiled using information provided by the Centers for Disease Control and Prevention National Diabetes Statistics Report.^2^ Prevalences of diabetes in the state of Ohio and northwest Ohio including Lucas County were compiled using reports published by the Ohio Department of Health^30^ and the Healthy Lucas County Coalition.^31^ Data on the national prevalence of spinal fusion surgeries were obtained from the report by Martin et al.^1^

### Tissue analysis

Prior to revision surgery for non-union, an informed consent was obtained from patients (total, 14 patients*)* to analyze removed tissue that occupied the posterolateral gutter between fused vertebrae. The procedure and the analysis had been approved by the Institutional Human Research Protection Review Board under the protocol IRB #202231. Specimens were analyzed for their macroscopic appearance followed by micro-CT using the μCT35 system (Scanco Medical AG, Bruettisellen, Switzerland), as previously described,^32^ to assess a microarchitecture of mineralized components found within tissue specimens. All mineralized components that presented any structural features in micro-CT scans were analyzed. Consequently, for nondiabetic patients, we analyzed 3 mineralized components obtained from 3 individuals and 2 separate components from each of the 4 remaining individuals. For diabetic patients, we analyzed 1 component obtained from 1 individual, 2 separate components obtained from each of 3 individuals, and 3 components from 1 remaining individual. Measurements were averaged for a given patient and plotted on graphs. Statistical analysis was performed on averaged values, as well as on nonaveraged values of 21 analyzed mineralized components. Statistical significance was maintained when using nonaveraged measurements, with *p*-values of .046, .003, and .023, respectively, for trabeculae number, thickness, and spacing.

### Statistical analysis

To assess whether diabetes contributes to the incidence of primary spinal fusion and revision surgery, a null hypothesis was adopted stating that the pre-existing diabetes has no influence on the incidence of either primary or revision surgery and was tested against a prevalence of diabetes in a community of the studied patient population. The presented study was retrospective in its nature and our data did not include a control group of patients with a spine condition but discharged without surgical intervention. Thus, we created virtual control groups (VCGs)^33^ in which the number of operated patients with the same indication for surgery was adjusted for the prevalence of diabetes in the Lucas County adult population. According to the Lucas County Health Assessment, the mean percentage of adult diabetics in this population for the years 2007–2017 was 13%^31^; therefore, the number of virtual patients in a given VCG was set to 13% of the number of actual patients for a given category. For instance, the VCG for 337 patients who underwent primary spinal fusion surgery was 44 (13% out of 337) and the VCG for 215 patients who underwent revision surgery was 28 (13% out of 215).

For the years 2007–2017, the period for the surgeries conducted at the local academic center, the mean Lucas County adult population was 321 756. The 337 patients who underwent primary surgery constituted a statistically representative sample of the Lucas County population at a 95% confidence level and 5.3% margin of error. Similarly, the 215 patients who underwent revision surgery also constituted a statistically applicable sample at a 95% confidence level and 6.7% margin of error. The data resulting from the retrospective observations regarding diabetes and surgical interventions were referenced to the numbers generated for the VCGs. Risk ratios with the corresponding CIs were calculated using 2 × 2 contingency tables with diabetic condition being absent or present for a specific indication in the studied group of patients versus VCG. The RR ratio and the corresponding CI for the diabetic versus control group sharing the same indication for primary and revision surgery are reported throughout the manuscript. Result for which the CI does not cross the value of 1 is regarded as being significant.^34^ Analyses of covariates were done separately for primary and revision surgeries, with diabetes being absent or present in groups of study patients with a specific covariate.

## Results

### Diabetes increased the risk of degenerative lumbar spine conditions requiring surgical intervention

Of all patients who underwent spinal surgeries between 2009 and 2017, 552 patients met the selection criteria for lumbar spinal fusion and were included in the study. Table 1 presents characteristics of the analyzed cohort. Of the selected patients, 25% were diabetic based on HbA1c levels and/or use of antidiabetic medications, which was almost 2-fold higher than diabetes occurrence in the community (13%).^2^^,^^30^^,^^31^ The average age was 54 (range, 22 to 87) years. The patient population consisted of 45% males and there was a tendency for more women than men being diabetic (26% vs 23%). The average BMI was 33.1 (range, 17–57) kg/m^2^, with higher mean value observed in the diabetic group (36.5 vs 31.9). Smoking and alcohol consumption were comparable between diabetic and nondiabetic patients. Data on patients’ ethnicity were not available.

**Table 1:** Characteristics of spinal fusion patients.

	**Total**	**Nondiabetic**	**Diabetic**
No. of patients	552	414 (75%)^a^	138 (25%)^a^
Age, yr	54 ± 13.7	52 ± 14.1	59 ± 11.3
BMI, kg/m^2^	33.1 ± 7.5	31.9 ± 7.1	36.5 ± 7.6
Gender, *n* (%)			
Male	250 (45%)^a^	192 (77%)^b^	58 (23%)^b^
Female	302 (55%)^a^	222 (74%)^b^	80 (26%)^b^
Smoking status, *n* (%)			
Never	206 (37%)^a^	148 (72%)^b^	58 (28%)^b^
Former	148 (27%)^a^	109 (74%)^b^	39 (26%)^b^
Current	198 (36%)^a^	157 (79%)^b^	41 (21%)^b^
Alcohol consumption, *n* (%)			
Yes	155 (28%)^a^	123 (79%)^b^	32 (21%)^b^
No	397 (72%)^a^	291 (73%)^b^	106 (27%)^b^

a Fraction of total number of patients.

b Fraction within category.

Among the 337 patients who underwent primary spinal fusion surgery, 21% were diabetic. Statistical comparison of this group of patients to a VCG representing an equal sample of the Lucas County diabetic population of 13% revealed that pre-existing diabetes is a predisposing factor for primary surgery (RR = 1.64; 95% CI, 1.16–2.31; *p* = .006) (Table 2). The most common indication for spinal fusion was degenerative disc, accounting for 47% of all cases (Table 2). Degenerative disc carried a modestly increased risk of surgery in those affected by diabetes (RR = 1.62; 95% CI, 0.98–2.66; *p* = .075). However, diabetic patients diagnosed with degenerative spondylolisthesis, a complex disorder resulting from degenerative changes in the intervertebral discs and facet joints, and accounting for 39% of all cases, were at significantly higher risk for primary surgery (RR = 1.76; 95% CI, 1.02–3.04; *p* = .052) (Table 2). In contrast, spondylolysis and isthmic spondylolisthesis, which are conditions associated with stress fracture of pars interarticularis, were far less frequent in diabetic versus nondiabetic patients, and accounted for only 17% of all cases. These conditions were also insignificant as indications for primary surgery in diabetic patients (RR = 1.33; 95% CI, 0.50–3.54; *p* = .773) (Table 2).

**Table 2:** Indications for spinal fusion primary surgery in diabetic vs nondiabetic patients, and calculated risk ratio (RR) vs a virtual control group (VCG).

**Indication**	**Total** ^**a**^	**Nondiabetic** ^**b**^	**Diabetic** ^**b**^	**RR [95% CI]** ^**c**^	***p*-Value** ^**d**^
All indications	337 (100%)	265 (79%)	72 (21%)	1.64 [1.16, 2.31]	.006
Degenerative disc^e^	159 (47%)	125 (79%)	34 (21%)	1.62 [0.98, 2.66]	.075
Degenerative spondylolisthesis	132 (39%)	102 (77%)	30 (23%)	1.76 [1.02, 3.04]	.052
Isthmic spondylolisthesis^f^	46 (17%)	38 (83%)	8 (17%)	1.33 [0.50, 3.54]	.773

a Fraction of total number of patients.

b Fraction of patients within category.

c VCG was established as described in Materials and Methods.

d Two-tailed Fisher’s exact probability test of RR.

e Includes degenerative prolapsed disc, degenerative scoliosis.

f Includes spondylolysis.

In conclusion, in the studied population, diabetes increased the risk for lumbar spinal fusion by 64%. Spine degenerative conditions were a more frequent indication for spinal fusion surgery among diabetic patients.

### In diabetic patients the risk of revision surgery exceeded the risk for primary surgery

In the analyzed cohort of the lumbar spinal fusion patients, there were 215 cases of revision surgery (Table 3). Diabetic patients constituted 31% of all revision cases in contrast to 21% of diabetic patients undergoing primary surgery. A comparison to the corresponding VCG indicated that pre-existing diabetes strongly predisposes to revision surgery (RR = 2.36; 95% CI, 1.58–3.52; *p* = 1.29 × 10^-5^). Adjacent segment disease was the most common indication and constituted 82% of all revision cases. Degenerative conditions, such as degenerative disc (RR = 2.00; 95% CI, 1.14–3.51; *p* = 0.015) and degenerative spondylolisthesis (RR = 3.17; 95% CI, 1.40–7.15; *p* = 0.004), were significant contributors to ASD in diabetes. Isthmic spondylolisthesis was not a significant factor leading to ASD, as only 1 out of all 12 cases was associated with diabetes (RR = 0.50; 95% CI, 0.05–4.81; *p* = .999). The risk of revision surgery due to pseudoarthrosis was significantly higher in diabetic patients (RR = 2.80; 95% CI, 1.12–7.02; *p* = .033). In general, the risk of revision surgery among diabetes was 44% higher than the risk for primary surgery, with spine degenerative conditions being a significant diagnostic indication for revision.

**Table 3:** Indications for spinal fusion revision surgery in diabetic vs nondiabetic patients, and calculated risk ratio (RR) vs a virtual control group (VCG).

**Indication**	**Total** ^**a**^	**Nondiabetic** ^**b**^	**Diabetic** ^**b**^	**RR [95% CI]** ^**c**^	***p*-Value** ^**d**^
All indications	215 (100%)	149 (69%)	66 (31%)	2.36 [1.58, 3.52]	1.29 × 10^-5^
Adjacent segment disease	176 (82%)	124 (71%)	52 (29%)	2.26 [1.45, 3.53]	2.35 × 10^-4^
Degenerative disc^e^	121	89 (74%)	32 (26%)	2.00 [1.16, 3.45]	.015
Degenerative spondylolisthesis	43	24 (56%)	19 (44%)	3.17 [1.40, 7.15]	.004
Isthmic spondylolisthesis^f^	12	11 (92%)	1 (8%)	0.50 [0.05, 4.81]	.999
Pseudoarthrosis	39 (18%)	25 (64%)	14 (36%)	2.80 [1.12, 7.02]	.033

a Fraction of total number of patients.

b Fraction of patients within category.

c VCG was established as described in Materials and Methods.

d Two-tailed Fisher’s exact probability test of RR.

e Includes degenerative prolapsed disc, degenerative scoliosis.

f Includes spondylolysis.

**Figure 1:**
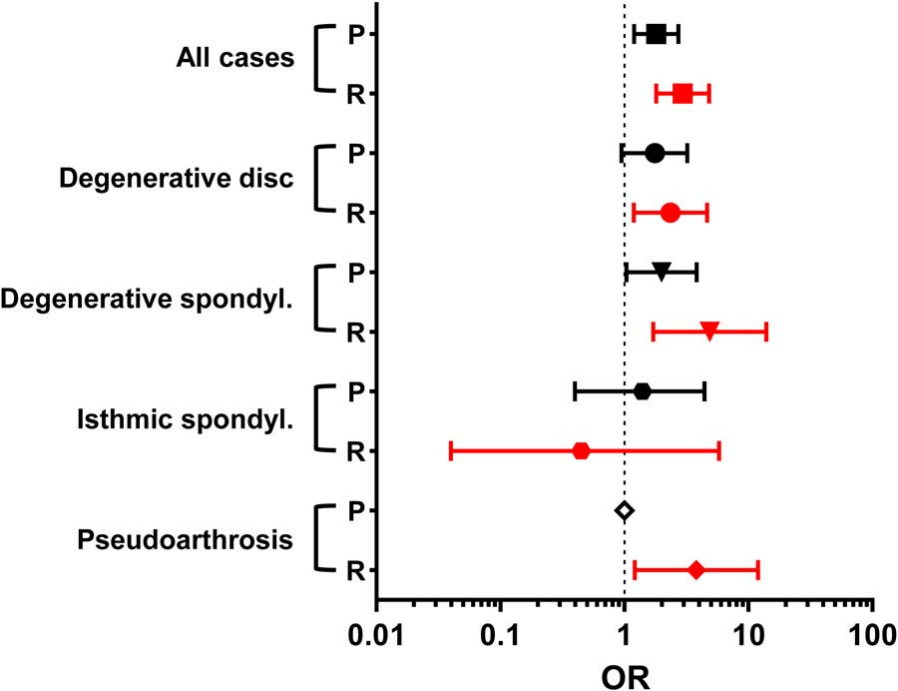
Odds ratios (OR) for diabetic patients’ spinal fusion indications in primary and revision surgery vs a control group (VCG). Abbreviations: P, primary surgery; R, revision surgery; spondyl., spondylolisthesis; VCG, virtual control group. Black - primary surgery (P); red - revision surgery (R).

The effects of covariates—namely, gender, age, BMI, smoking, and use of alcohol—on the incidence of primary and revision surgery among diabetic patients are shown in Table 4. Gender was not a predisposing factor for primary surgery; however, a trend towards a higher risk for revision surgery was observed among women with diabetes (RR = 1.49; 95% CI, 0.98–2.24; *p* = .076). The RR for primary and revision surgery was not dependent on the mean age of diabetic vs nondiabetic patients. A BMI over 25 kg/m^2^ was, by itself, a significant risk factor for primary and revision surgery (primary RR = 2.69; 95% CI, 1.03–7.01; *p* = .031; revision RR = 3.02; 95% CI, 1.02–8.93; *p* = .024). However, since diabetes and obesity are often inherently linked, it was not possible to establish the true contribution of BMI to the need for either surgery. Incidentally, current smokers vs nonsmokers had a lower risk for revision surgery (RR = 0.59; 95% CI, 0.36–0.97; *p* = .034); however, a comparison of current vs former smokers and former vs nonsmokers showed that there was no significant difference with regard to the incidence of primary or revision surgery. The status of alcohol use among diabetics was not a determining factor for either surgery. Figure 1 shows a graphical presentation of odds ratios (ORs) in diabetic patients to develop spine degenerative conditions predisposing to either primary or revision surgery.

**Table 4:** Odds ratio (OR) and risk ratio (RR) of covariates for primary and revision spinal fusion surgery in diabetic vs nondiabetic patients.

**Factor**	**Surgery**	**Relation**	**RR [95% CI]**	***p*-Value**
Gender	Primary	F vs M	0.95 [0.63, 1.44]	.893
	Revision	F vs M	1.49 [0.98, 2.24]	.076
Age	Primary	>59 vs <59 yr	1.12 [0.74, 1.69]	.688
	Revision	>59 vs <59 yr	0.88 [0.59, 1.31]	.557
BMI	Primary	>25 vs <25 kg/m^2^	2.69 [1.03, 7.01]	.031
	Revision	>25 vs <25 kg/m^2^	3.02 [1.02, 8.93]	.024
Smoking	Primary	C vs NS	0.85 [0.52, 1.38]	.537
	Revision	C vs NS	0.59 [0.36, 0.97]	.034
Smoking	Primary	C vs FS	0.94 [0.54, 1.63]	.862
	Revision	C vs FS	0.63 [0.37, 1.07]	.124
Smoking	Primary	N vs FS	1.11 [0.67, 1.84]	.746
	Revision	N vs FS	1.07 [0.67, 1.70]	.855
Alcohol	Primary	Y vs N	0.84 [0.52, 1.34]	.473
	Revision	Y vs N	0.73 [0.43, 1.23]	.238

Abbreviations: C, current smokers; F, female; FS, former smokers; M, male; N, not drinking alcohol; NS, never smokers; Y, drinking alcohol.

### Analysis of paravertebral tissue removed during revision surgery

**Figure 2:**
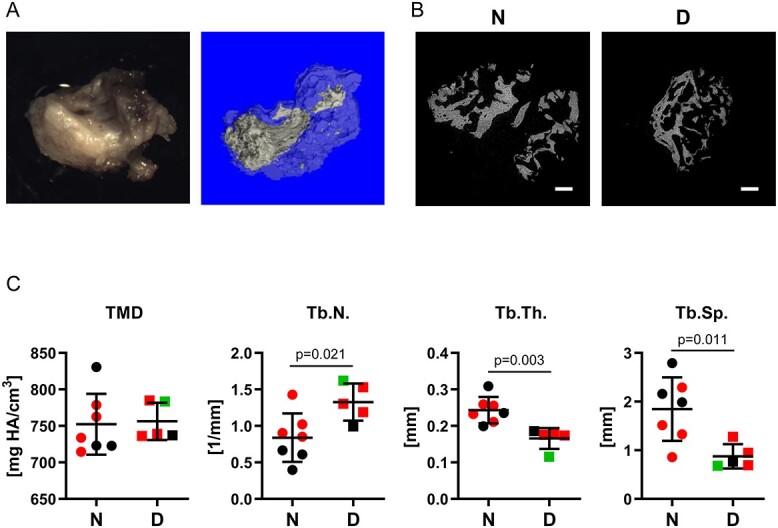
Analyses of tissue specimens retrieved during spinal fusion revision surgery. A: Bright-light photography (left panel) and cross-section of micro-CT rendering (right panel; gray area represents mineralized component) exemplify tissue isolated from the spinal fusion bed during revision surgery. B: Examples of micro-CT renderings of mineralized components present in tissue specimens isolated from a nondiabetic patient (N) and from a diabetic patient (D). Bars represent 100 μm. C: Micro-CT analysis of mineralized components present in tissue specimens isolated from nondiabetic patients (N) and from diabetic patients (D). Statistical differences were calculated using unpaired Student’s *t* test. Black symbols represent measurements of single mineralized component obtained from an individual patient. Red and green symbols represent the average of measurements of 2 or 3 mineralized components obtained from an individual patient, respectively. Abbreviations: Tb.N., number of struts; Tb.Sp., spacing between struts; Tb.Th., strut thickness; TMD, tissue mineral density (a measure of hydroxyapatite amount in mineralized component); HA - hydroxyapatite.

Fourteen specimens of paravertebral tissue from the posterolateral gutter located at the fusion bed were collected and processed for analysis of bone nodule microarchitecture. Specimens were obtained from 7 nondiabetic (2 male and 5 female) and 7 diabetic (4 female and 3 male) patients, who were matched on BMI to limit variables confounding the analysis (Table 5). Collected specimens contained newly formed tissue at the site of the original spinal fusion procedure, which was expected to result in developing bone-like mineralized tissue to provide support for orthopedic hardware coupling the adjacent vertebrae. Macroscopic examination and micro-CT analysis showed that paravertebral tissue samples consisted mostly of a mixture of connective and adipocytic tissue and various amounts of calcified material (Figure 2A). All non-union specimens collected from nondiabetic patients contained components of mineralized tissue showing some degree of apparent bone-forming activity. Tissues collected from 2 diabetic patients (1 male and 1 female) did not contain any trace of mineralized component, with the 5 other tissue samples showing low-grade bone formation (Figure 2B and C). Micro-CT analysis of the mineral composition of bone deposits present in tissue samples showed no difference in the content of hydroxyapatite in diabetic vs nondiabetic groups, indicating no defect in mineralization. However, there were significant differences between groups in the microarchitecture of bone present in these specimens. Newly formed bone in diabetic specimens was mainly composed of densely distributed thin struts (higher Tb.N.), while bone structural components present in nondiabetic specimens were less numerous, albeit much thicker (higher Tb.Th.), and in consequence, less densely distributed (lower Tb.Sp.) (Figure 2B and C). These morphologic features suggested that, in diabetes, a process of new bone formation differs from that in nondiabetic conditions, and newly formed bone is of lower structural quality.

**Table 5:** Donors of paravertebral tissue collected during revision surgery for non-union.

**Status**	**HbA1c, %**	**Age, yr**	**BMI, kg/m** ^**2**^	**No. of patients (gender)**
N	n.d.	47 ± 9	35 ± 11	7 (5 F, 2 M)
D	6.7 ± 1.2	61 ± 6	35 ± 5	7 (4 F, 3 M)

Abbreviations: D, diabetic; F, female; HbA1c, glycated hemoglobin; M, male; N, nondiabetic; n.d., no data.

## Discussion

This observational study was conducted to determine the predisposition of diabetic individuals undergoing lumbar spinal fusion surgery to complications, other than infections, requiring revision surgery. In general, diabetes predisposes to degenerative spine pathological conditions requiring surgical intervention. The presented evidence indicates that diabetes increases the risk for revision surgery due to the development of ASD and non-union complications to levels exceeding the overall risk of primary surgery. It has also been demonstrated that the microarchitecture of bone components isolated from paravertebral tissue removed during revision surgery was inferior in diabetic vs nondiabetic patients.

Our analysis showed that diabetes carries an RR of 2.36 for revision surgery, which is 44% greater than the RR of 1.64 for primary lumbar spinal fusion surgery. The study population consisted of 25% diabetics, which is 2-fold higher than expected based on diabetes prevalence in northwest Ohio (13%) and the national average (11.3%).^2^^,^^30^ As compared with primary surgery, there was a 30% increase in the number of diabetic individuals undergoing revision surgery.

In line with other studies,^6^^,^^10^^,^^11^ we found intervertebral disc degeneration and degenerative spondylolisthesis as being disproportionally more frequent indications for primary spinal fusion surgery in patients with diabetes than was expected from the prevalence of this disease in the studied population. In this study, we showed that spine degenerative conditions are major contributors to the development of ASD in diabetic individuals requiring revision surgery (RR = 2.26; 95% CI, 1.45–3.53). This is consistent with the number of human and animal studies indicating that diabetes contributes to disc degeneration by changing its composition and matrix biomechanical properties, and that these changes are, at least in part, due to a diabetes-associated increase in low-grade inflammation.^9^^,^^35^ Our study confirms that isthmic spondylolisthesis was not a significant indication for primary surgery,^36^ and shows that this condition is not a significant contributor to the development of ASD in diabetes.

The RR of 2.80 (95% CI, 1.12–7.02) for pseudoarthrosis among diabetic patients was also far greater than expected based on the proportion of diabetics vs nondiabetics in the general population and suggested a defect in new bone formation. It has been shown that diabetes affects bone formation via a complex mechanism affecting osteoblast activity, including a toxic effect of glucose and a local increase in proinflammatory cytokines, such as TNF-α, IL-1β, IL-6, and IL-18.^5^^,^^37-39^ Consistent with these findings, it has been demonstrated in a rat model of spinal fusion that new bone formation negatively correlated with blood glucose levels and elevated levels of proinflammatory cytokines,^40^ as well as with an altered response of mesenchymal progenitors to pro-osteoblastic signaling.^41^ Of note, a retrospective analysis of 16 085 cases of total hip and total knee arthroplasty showed that high glucose levels before surgery significantly increased the risk of aseptic loosening of implants, suggesting a defect in healing of the bone-implant interface.^42^

Our analysis of newly formed bone present in the paravertebral spinal fusion bed showed significant differences in bone microarchitecture between diabetic and nondiabetic individuals, which is consistent with the affected activity of bone-forming osteoblasts. Diabetes correlated with poorly developed bone tissue consisting of numerous thin struts. If such structure is also present in diabetic patients with successful fusion, it may lead to compromised support for fused vertebrae and consequently to long-term complications. To our knowledge, this is the first analysis of the microstructure of calcified components in human paravertebral tissue isolated from the non-union fusion bed and correlated with diabetes status.

We found a positive association between BMI and lumbar spinal fusion, both in primary and revision surgery, which is consistent with a number of other studies.^16^^,^^25^^,^^26^ Gender was not a risk factor for the primary surgery. However, a tendency of increased risk for revision surgery was observed in women. With an absence of more information on female patients, including bone health and estrogen status, this observation cannot be interpreted. Smoking had been previously indicated as a risk factor for revision surgery.^43^ Surprisingly, in our study, current smoking correlated inversely with revision surgery. Although this may suggest beneficial effects of smoking, it is more likely that smoking in our population was associated with alleviating pain and stress due to the condition of the spine, and perhaps avoiding or delaying revision surgery. Notable, in our study, 36% of patients were current smokers, which is more than twice the 14% smoking rate in northwest Ohio.^31^

There are several strengths and limitations of this study. The strengths include a specific insight into a community where compararable patients reside in the same geographic region of northwest Ohio and come from a modest-income neighborhood surrounding the medical center where the study was conducted. This approach, which relies on the analysis of local circumstances affecting the local community, may offer an extra level of information to comparisons made in larger and more diverse populations. In addition, spinal fusion surgeries were performed in the same hospital by a single orthopedic surgeon, which minimized variables associated with postoperative care and different surgery techniques. However, the study has the inherent limitation of being a retrospective study and dependent on data entered into a clinical database with some incomplete information, including the type of diabetic disease (eg, insulin-dependent vs insulin-independent) and bone health status (eg, diagnosis of osteopenia or osteoporosis), which would allow for better determination of risk factors. Another limitation consists of the relatively low number of patients, which precluded correlation analysis of fusion technical aspects (eg, long vs short, or posterior lumbar interbody vs posterolateral fusion) and the development of pseudoarthrosis in diabetic patients, as well as clinical indications for spinal fusion and use of certain medications. It is well established that medications, including antidepressants and painkillers, significantly affect new bone formation and bone healing. Available medical records indicated that all patients were taking a number of prescription drugs, including selective serotonin reuptake inhibitors (SSRIs) and opioids, which are known to increase spinal fusion complications.^44-46^ A multicenter prospective study with larger patient numbers and a wider socioeconomic status should address some of these questions.

In summary, we have shown that there is a significant causal link between diabetes and increased rate of postoperative lumbar spinal fusion complications requiring revision surgery. Identifying variables affecting spinal fusion success in diabetics may provide a platform to develop a model to predict complications and intervention. As the population of patients who live with diabetes increases, it is inevitable that a proportion of patients undergoing spinal fusion will experience concurrent diabetes that consequently will increase the risk of non-union complications. Glucose homeostasis is of critical importance to human health and inappropriate blood glucose levels affect a multitude of processes at the cellular level. Therefore, it is imperative to continue the identification of epigenetic imprints resulting from increased blood glucose, which, in consequence, affect bone-forming cells in the context of bone formation at the spinal fusion site. In addition to the already suggested approaches to decrease HbA1c and glucose levels during the pre- and postoperative period,^47^ manipulation of the microenvironment supporting new bone formation at the fusion site, either endogenous or with the help of bone and cellular grafts,^48^^,^^49^ might be a viable option to increase spinal fusion success rate in diabetic patients.

## Author contributions

Claire Wilson (Data curation, Formal analysis, Investigation, Methodology, Writing – original draft [equal]), Piotr J. Czernik (Data curation, Investigation, Methodology, Visualization, Writing—original draft, Writing—review & editing [equal]), Hossein Elgafy (Conceptualization, Methodology, Resources, Validation, Writing—review & editing), Sadik Khuder (Data curation, Formal analysis), Kevin Serdaherly (Data curation, Methodology), Andrea Rowland (Data curation, Methodology), Beata Lecka-Czernik (Conceptualization, Data curation, Formal analysis, Funding acquisition, Project administration, Supervision, Writing—original draft, Writing—review & editing).

## Funding

This work was supported by grants to B.L.-C. from the National Institutes of Health R01AG071332 and the American Diabetes Association Innovative Basic Science Award no. 1-19-IBS-029.

## Conflicts of interest

All authors have no competing interests to declare.

## Data availablility

All data are available upon request.
